# Genetic Regulation of Maternal Oxytocin Response and Its Influences on Maternal Behavior

**DOI:** 10.1155/2016/5740365

**Published:** 2016-10-31

**Authors:** Divya Mehta, Valsamma Eapen, Jane Kohlhoff, Antonio Mendoza Diaz, Bryanne Barnett, Derrick Silove, Mark R. Dadds

**Affiliations:** ^1^School of Psychology and Counseling, Faculty of Health, Institute of Health and Biomedical Innovation, Queensland University of Technology, Kelvin Grove, QLD, Australia; ^2^School of Psychiatry, University of New South Wales, Sydney, NSW, Australia; ^3^Academic Unit of Child Psychiatry, South West Sydney Local Health District and Ingham Institute, Sydney, NSW, Australia; ^4^Karitane, Villawood, NSW, Australia; ^5^Sydney Child Behaviour Research Clinic, School of Psychology, University of Sydney, Sydney, NSW, Australia; ^6^Academic Mental Health Unit and Psychiatry Research and Teaching Unit, Sydney South West Local Health District, Liverpool Hospital, Sydney, NSW, Australia

## Abstract

We interrogated the genetic modulation of maternal oxytocin response and its association with maternal behavior using genetic risk scores within the oxytocin receptor (OXTR) gene. We identified a novel SNP, rs968389, to be significantly associated with maternal oxytocin response after a challenging mother-infant interaction task (Still Face Paradigm) and maternal separation anxiety from the infant. Performing a multiallelic analysis across OXTR by calculating a cumulative genetic risk score revealed a significant gene-by-environment (G × E) interaction, with OXTR genetic risk score interacting with adult separation anxiety to modulate levels of maternal sensitivity. Mothers with higher OXTR genetic risk score and adult separation anxiety showed significantly reduced levels of maternal sensitivity during free play with the infant. The same G × E interaction was also observed for the extended OXTR cumulative genetic risk score that included rs968389. Moreover, the extended cumulative OXTR genetic risk score itself was found to be significantly associated with maternal separation anxiety as it specifically relates to the infant. Our results suggest a complex montage of individual and synergistic genetic mediators of maternal behavior. These findings add to specific knowledge about genetic regulation of maternal oxytocin response in relation to maternal adjustment and infant bonding through the first few months of life.

## 1. Introduction

Humans are social beings who are biologically tuned to form selective and enduring bonds with other individuals. These social bonds provide protection and caregiving and offer comfort during times of distress [1]. A plethora of genetic, epigenetic, behavioral, neurological, and cognitive processes work in unison to form these meaningful bonds that are essential for human survival [2]. Several studies have examined aspects of the neuroendocrinological basis for social behavior and human bonding. Oxytocin is a neurohormone implicated in the human affiliation system, particularly involving social behaviors, parent-infant bonding [3, 4], and maternal caregiving behavior [5]. Studies in humans [6–8] and rodents [5, 9] have demonstrated the role of oxytocin in maternal caregiving behavior, with higher levels observed in securely attached mothers after interaction with their infants. Mothers with the highest oxytocin levels during parturition demonstrate more attentive maternal behaviors in relation to the infant, including gaze, vocalization, positive affect, affectionate touch, frequent checking of the baby, and attachment-related thoughts concerning the infant following birth [10]. Also, significant interindividual differences are described in the maternal oxytocin response after interaction with the infant, characteristics which in turn may predict differences in the quality and extent of maternal care [11, 12]. Further, mothers with secure attachment styles show an increased oxytocin response following interaction with their infants, exhibiting greater activation of the brain reward regions and the oxytocin-associated hypothalamus/pituitary region [6]. Our group and other researchers have found an association between lower plasma oxytocin levels in the postpartum period and symptoms of adult separation anxiety (ASA) and depression in mothers; in addition, these mothers exhibited anxious attachment styles [13]. In the same study, ASA mediated the association between anxious attachment and depression, while depressed mood mediated the relationship between separation anxiety and low oxytocin levels [13].

The involvement of oxytocin in regulating the mother's responsive engagement with her infant has been studied using the Still Face Paradigm (SFP) [14]. SFP is an experimental paradigm initially developed to examine an infant's reaction to a state of unresponsiveness in the mother, and the paradigm has subsequently been extended to the study of a myriad of related research questions including aspects of mother-infant interaction [15] and the creation of an emotional challenge for the infant-mother dyad [16, 17]. In a study examining change in plasma oxytocin levels following interactions with the infant during the SFP condition [16], mothers with a reduced oxytocin response demonstrated a significant decrease in their gaze toward their infants during periods of infant distress, while such changes were not observed in mothers with an increased oxytocin response.

Oxytocin, however, may be associated with both positive and adverse outcomes depending on the level of nurturance or adversity in the early environment of an individual [3]. For instance, recent genetic studies have provided evidence of gene-environment (G × E) interactions, such that the SNPs within the oxytocin receptor gene (OXTR) interact with the environment to confer risk for mental disorders and other behavioral traits. Interaction of the OXTR genotypes with positive behaviors such as empathy [18] and trust [19] or, conversely, with adverse environments such as exposure to abuse [20, 21] shapes either lower or greater risk for depression and anxiety, respectively.

Genetic variability within the OXTR gene may modulate risk for psychiatric and behavioral symptoms, and this may be driven by a combination of cumulative genetic effects across the OXTR locus. Cumulative genetic risk score across OXTR, CD38, and AVPR1a, previously associated with psychopathology, sociability, and caregiving of the mother, has been shown to interact with trauma-exposure in children to predict higher levels of PTSD, anxiety disorders, conduct disorders, and ADHD [22], further supporting the role of OXTR gene-environmental interactions.

The overarching aim of the study was to investigate relations involving blood oxytocin levels, aspects of the oxytocin gene, and maternal caregiving behavior. Specifically, we aimed to evaluate (a) the genetic mediators of maternal oxytocin response following a challenging mother-infant interaction task (i.e., SFP); test if the same genetic mediators would be associated with (b) maternal behavior following a mother-infant separation and interrogate (c) whether the genetically mediated maternal behavior was driven by separation anxiety. We hypothesized that mothers with a genetic predisposition to decreased maternal oxytocin response would be more likely to exhibit reduced maternal caregiving behavior, and this relationship would be mediated by separation anxiety.

## 2. Materials and Methods

### 2.1. Study Participants

The study sample comprised 127 participants, selected from a larger pool of pregnant women (*n* = 668) attending a general hospital antenatal clinic, the details being provided in Eapen et al., 2014 [13]. Briefly, 668 women were screened in pregnancy using the Adult Separation Anxiety Questionnaire (ASA-27) to assess eligibility for a longitudinal study investigating maternal separation anxiety, oxytocin, and bonding over the transition to parenthood. The antenatal sample of women was sampled so as to achieve a structured group including a balanced number of participants with and without current separation anxiety symptoms as indicated by the ASA-27. The study cohort of 127 women had an almost equal number of women with and without ASA, with 27 more than the required 100 subjects, to allow for attrition of the sample over time. From this sample of 127 women, genotypes for OXTR SNPs were available for a random subset of 96 women that were included in this study. All analyses described here were performed on this subset of 96 women. At the time of recruitment, all women were aged over 18 years, English-speaking, and less than 38 weeks' gestation with a singleton infant. Women provided consent to participate in a longitudinal study and genetic analysis investigating maternal separation anxiety, maternal bonding, and related experiences. Demographic details of women included in the study are provided in Table 1.

#### 2.1.1. Procedure

Prenatally (averaging 30 weeks' gestation), participants completed self-report questionnaires including the Adult Separation Anxiety Questionnaire (ASA-27) [23]. For the postnatal stage of the study (averaging 3 months postpartum), participants again completed the ASA-27 as well as the Maternal Separation Anxiety Scale (MSAS) [24] and then took part in the Still Face Paradigm [14] in addition to providing blood samples for oxytocin measurements and DNA for genotyping.

### 2.2. Questionnaires and Mother-Infant Observational Procedure

*(i) The Adult Separation Anxiety Checklist Scale (ASA-27)*. The ASA-27 is a self-report questionnaire assessing separation anxiety symptoms in adulthood [23]. The ASA-27 contains 27 items, each answered on a four-point scale. Scores can be summed to yield a total score, with higher scores indicating greater separation anxiety symptom severity. The measure has sound internal consistency (Cronbach's alpha of 0.95). To allow comparisons with other studies examining anxiety in pregnancy and the unique contribution of separation anxiety to maternal bonding and related experiences, participants were allocated to the “separation anxiety group” if they scored over the threshold (i.e., total ASA-27 score ≥ 22) [25] for clinically significant symptoms or to the “without separation anxiety group” if they scored below threshold [13]. The cut-off threshold for determining ASA was achieved by comparing the ASA-27 with a structured clinical interview for separation anxiety disorder (Adult Separation Anxiety Semistructured Interview: ASA-SI) that revealed a high level of concordance suggesting that the questionnaire is an adequate alternative measure of ASA [25].

*(ii) Maternal Separation Anxiety Scale (MSAS)*. Maternal separation anxiety is characterized by an unpleasant emotional state resulting from a separation experience from her child, manifested by feelings of worry, sadness, or guilt in the mother [24]. The MSAS is a 35-item scale composed of 3 subscales assessing a mother's concerns about separation from her child. Subscale 1 includes items related to feelings of worry, sadness, and guilt surrounding a separation event and attitudes about exclusive maternal care (maternal care giving behavior as it specifically relates to the infant including separation from the infant). Subscale 2 measures maternal perceptions of the child's response to separation. Subscale 3 measures maternal feelings about employment-related separation concerns [24].

*(iii) Still Face Paradigm (SFP)*. The Still Face Paradigm (SFP) [7, 24] is a robust and extensively used mother-infant interaction procedure comprising consecutive episodes of “free play” (3 minutes), maternal “still face” (90 seconds), and “reunion” (2 minutes) used in this study to present a “challenging” task for the mother-infant dyad. A trained coder, blind to participants' questionnaire scores and genetic subtyping, rated maternal behaviors (“maternal sensitivity”) during the “free play” and “reunion” episodes of the Still Face Paradigm using the Global Scales for Mother-Infant Interaction [26]. Specifically, maternal sensitivity was coded using the good-poor dimension, rating the extent to which the mother responds to her infant's cues by appropriately adjusting to the infant's behavior, responding to infants' signals, and measuring warmth and acceptance. For the SFP, ten percent of cases were recoded by a second coder who was also blind to participant scores on all study variables. The intraclass correlation coefficient for the two coders for maternal sensitivity was .73.

### 2.3. Experimental Procedures

*Participants' Plasma Oxytocin Extraction and Radioimmunoassay (RIA)*. Detailed experimental protocols are given in Eapen et al., 2014 [13]. The radioimmunoassay (RIA) method used in this study was developed using independently derived antisera and independently conducted validations and is one of the best available methods for plasma oxytocin estimation [27]. Blood samples were taken before (T1) and after (T2) the Still Face Paradigm, collected into vacutainer tubes, and then centrifuged at 3000 RPM for 10 min. Plasma supernatants were pipetted and stored at −20°C until extraction was performed. At the time of extraction, 20 mg heat-activated (700 uC) LiChroprep Si 60 (Merck) in 1 mL distilled water was added to each sample, mixed for 30 minutes, and centrifuged. The pellet was washed with distilled water and 0.01 N acidic acid and then mixed with 60% acetone to elude the neuropeptide, and evaporated extracts were kept at −20°C. The 0.05 mL of assay buffer was added and oxytocin assessed using a highly sensitive and specific radioimmunoassay. To each aliquot, 0.05 mL antibody and 0.01 mL l-labeled tracer were added and after an incubation period of 3 days unbound radioactivity was precipitated by activated charcoal. All evaporated plasma extracts to be compared were treated identically to reduce any technical artefacts during the experiment. The detection limit is in the 0.5 pg/sample range and antiserum cross-reactivity of less than 0.7%. Samples were analyzed at the Max Plank Institute of Psychiatry, Munich, Germany [28].

*OXTR Sequencing*. OXTR sequencing was performed by AGFR Genomics Facility using their standard protocols. Sequencing was performed using MiSeq Reagent Kit v2 (500 cycles) 2 × 250 bp paired end reads. Image analysis was performed in real time by the MiSeq Control Software (MCS) v2.5.0.5 and Real Time Analysis (RTA) v1.18.54, running on the instrument computer. Then the Illumina bcl2fastq 1.8.4 pipeline was used to generate the sequence data. The data generated met the AGRF quality standards. A total of 12 million reads were generated with >92% aligning to the human genome.

### 2.4. Statistical Procedures

Raw genotype data were exported and converted to standard genotype calls. All data analysis was performed using plink and R. Linear regression models in R were fitted for covariates of age and ethnicity. Quality checks removed ambiguous SNPs, SNPs with minor allele frequency (MAF) < 10%, and deviations from Hardy Weinberg equilibrium (HWE *p* value < 0.000001). All models used the additive SNP models. This allowed a total of 19 OXTR SNPs that were used in further analysis. Applying a multiple testing correction, this corresponds to a corrected *p* value of 0.05/19 = 0.00263^*∗*^ Bonferroni threshold.

Imputation was performed using the minimac3 via the Michigan imputation server (https://imputationserver.sph.umich.edu/index.html). Filtering was done for MAF of 10% and R^2^ of 0.20. The 3 OXTR SNPs previously reported by Feldman et al., 2014, were extracted from the imputed filtered dataset [22]. In line with Feldman et al., a cumulative genetic risk score for OXTR was calculated across rs53576, rs2254298, and rs1042778 (G) which has been previously associated with parental sensitivity and parent-child reciprocity. For each OXTR SNP, the genotypes were divided into “high risk” and “low risk” groups as described by Feldman et al., 2014. The cumulative genetic risk was computed for each individual by combining the number of high-risk genotypes on each of the SNPs and summing it across all the OXTR SNPs. An extended cumulative genetic risk score across the 3 OXTR SNPs and rs968389 was calculated using the allele associated with lower oxytocin levels as the risk group. The clinical cut-off (≥22) for the ASA-27 (adult separation anxiety) was used to group the environmental factor (E) adult separation anxiety as binomial [25]. For MSAS, analysis was performed for each individual subscale as well as for the total MSAS score. Depending on the variable of interest, linear or logistic regressions were performed for the main and gene-environmental interaction models, after correction for covariates, using standard glm functions in R. Coefficients of determination (adjusted R^2^) were extracted to indicate the proportion of the variance in the dependent variable explained by the independent variable.

To confirm that the SNP identified in this study had functional consequences, we queried previous databases and publications for functional evidence. We used the Genotype-Tissue Expression (GTEx) project data (http://www.gtexportal.org/home/) and the UK Brain Expression Consortium data (http://www.braineac.org/) to evaluate functional evidence for the SNP identified in this study. These databases allow assessing genetic variation within the genomes by analyzing global RNA expression within individual tissues and identifying variations in gene expression that are highly correlated with genetic variation, also known as expression quantitative trait loci, or eQTLs.

## 3. Results

### 3.1. Genetic Modulation of Oxytocin Levels after a Still Face Paradigm and Association with Maternal Separation Anxiety from the Infant

**Figure 1:**
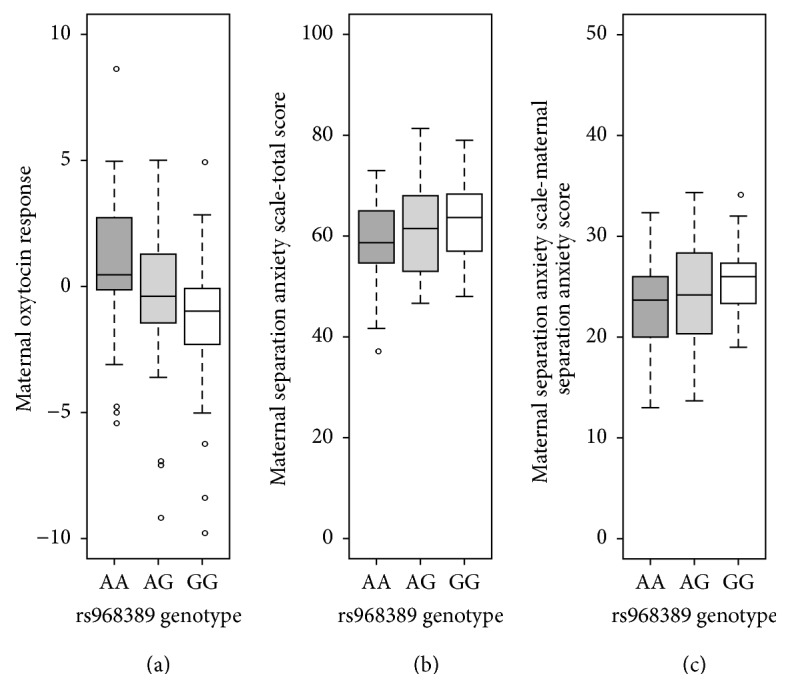
OXTR and maternal oxytocin response, MSAS total scores, and maternal separation anxiety. SNP rs968389 within the OXTR gene was significantly associated with maternal oxytocin response (change in oxytocin levels following the SFP) ((a) *p* = 0.0023^*∗∗*^,  R^2^ = 9.1%), total MSAS scores ((b) *p* = 0.033^*∗*^,  R^2^ = 3.9%), and maternal separation anxiety from the infant ((c) *p* = 0.014^*∗*^,  R^2^ = 4.9%). *∗* indicates statistical significance at *p* values <0.05, while *∗∗* indicates *p* < 0.01.

First, we sought to identify SNPs within the OXTR gene that significantly regulated maternal oxytocin response (difference in oxytocin levels before and after the SFP). An association analysis was performed using sequencing data that was available for a total of 19 independent OXTR SNPs. Of the 19 tested SNPs, one SNP within the OXTR, rs968389, was significantly associated with maternal oxytocin response (*p* = 0.0023^*∗∗*^, R^2^ = 9.1%), even after Bonferroni correction for multiple testing (Figure 1(a)) and after correcting for covariates of age and ethnicity (*p* = 0.00259^*∗∗*^).

Given the known association between oxytocin and maternal caregiving behavior, next, we tested the relationship between this SNP and maternal behavior specifically related to the care of the infant including separation anxiety as measured by the MSAS subscales. The rs968389 was significantly associated with the total MSAS scores (*p* = 0.033^*∗*^, R^2^ = 3.9%) and specifically with maternal care behavior including when separating from the infant (*p* = 0.014^*∗*^, R^2^ = 4.9%; see Figure 1). No significant association was observed for maternal perception of the child's response to separation nor maternal feelings about employment-related separation concerns (*p* > 0.05).

### 3.2. Cumulative Genetic Risk of Oxytocin Receptor Gene and G × E on Maternal Sensitivity

In line with Feldman et al. [22], we calculated the cumulative genetic risk score across the 3 OXTR SNPs used in the Feldman study. The cumulative genetic risk score of OXTR was not associated with the total MSAS scores nor with the subscales of maternal separation anxiety from the infant, maternal perception of the child's response to separation, or maternal feelings about employment-related separation concerns (*p* > 0.05). Nevertheless, mothers with a higher OXTR cumulative genetic risk score showed significantly decreased levels of observed maternal sensitivity as measured during the free play and the reunion episodes of the SFP (*p* = 0.038^*∗*^, R^2^ = 8.4%).

**Figure 2:**
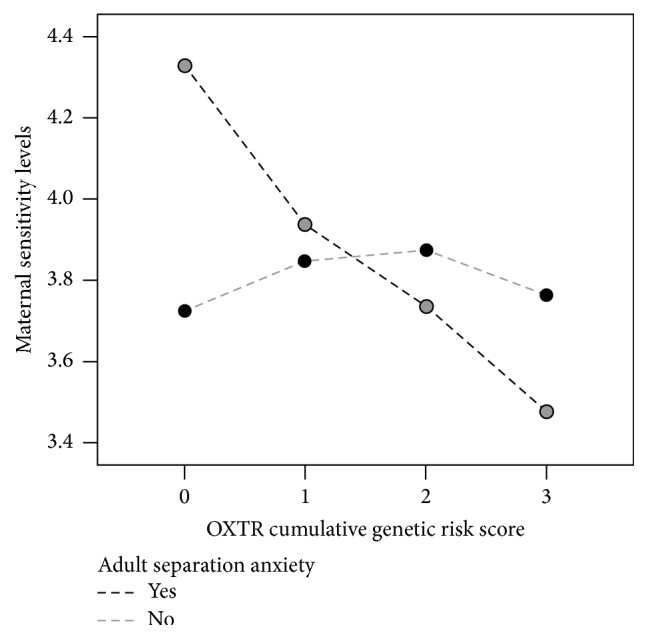
Significant G × E observed for adult separation anxiety and OXTR cumulative genetic risk score on maternal sensitivity. Mothers with higher OXTR genetic risk score and adult separation anxiety tend to have reduced levels of maternal sensitivity (*p* = 0.007^*∗∗*^); group means are depicted to indicate the trend. *∗∗* indicates *p* < 0.01.

We next questioned whether the association between the genetic risk score and maternal sensitivity was driven by anxiety, using assignment to high or low adult separation anxiety group as measured by the ASA-27. We tested “evocative” gene-environment interaction [29] using ASA and found a significant G × E association between the OXTR cumulative genetic risk score and adult separation anxiety assignment on observed maternal sensitivity during free play of the SFP (*p* = 0.007^*∗∗*^, R^2^ = 14%, Figure 2).

Next, we calculated an extended cumulative genetic risk score across the 3 OXTR SNPs and rs968389, the SNP that we identified to be significantly associated with maternal oxytocin response after the SFP, as shown above. A significant G × E association was observed between OXTR extended cumulative genetic risk score and adult separation anxiety assignment on the ASA-27 on observed maternal sensitivity during free play (*p* = 0.035^*∗*^, R^2^ = 5.4%) of the SFP, similar to that observed above with the cumulative risk score from Feldman and colleagues. In addition, the extended OXTR cumulative genetic risk score was also significantly associated with total MSAS scores (*p* = 0.039^*∗*^, R^2^ = 3.6%) and the subscale measuring maternal separation anxiety from the infant (*p* = 0.022^*∗*^, R^2^ = 3.6%); however, this association was mainly driven by SNP rs968389.

## 4. Discussion

In this study, we interrogated the genetic and environmental modulation of maternal oxytocin response and tested its association with observed maternal behaviors using single and multilocus genetic risk scores within the OXTR gene. Our results suggest that maternal oxytocin response is genetically regulated and we identified a SNP within the OXTR gene rs968389 that was significantly associated with changes in oxytocin levels following the SFP. Additionally, this same SNP was found to be associated with attitudes about exclusive maternal care and maternal separation anxiety as it relates specifically to the infant, highlighting its role in maternal behavior. While most mothers are likely to experience some anxiety at separation from their infant, some women experience intense apprehension and concern [30], resulting in detrimental outcomes for maternal psychological functioning, infant development, and overall family functioning [24]. As expected, mothers with reduced oxytocin response had increased levels of maternal separation anxiety from the infant. To the best of our knowledge, this is the first report indicating this SNP in maternal oxytocin response and maternal behavior.

Using the publicly available GTeX database [31] and the Braineac database of the UK Brain Expression Consortium (http://www.braineac.org/), we found that rs968389 was significantly associated with gene expression of the OXTR gene within the caudate (basal ganglia) brain tissue (*p* value = 1.7*E* − 8) as well as the frontal cortex (*p* value = 8.7*E* − 7) among others, providing evidence of functional consequences of the SNP (see Supplementary Figures 1a and 1b in Supplementary Material available online at http://dx.doi.org/10.1155/2016/5740365). Moreover, the identified SNP rs968389 is also in high LD (R^2^ = 0.9) with another SNP rs237897, and this SNP was recently reported to significantly influence DNA methylation levels for two CpG sites (*p* < 0.05) within the OXTR gene [32]. Gene regulatory mechanisms are constantly influenced by environmental factors that have the ability to interact with the underlying genotype to dynamically regulate protein activity, thereby influencing oxytocin signaling processes.

We extended the work of Feldman and colleagues by using a similar approach and building a cumulative genetic risk score across four SNPs within OXTR, previously associated with social and maternal behavior [22]. In addition to classical gene-environment interactions, “evocative” gene-environment interactions that explain how heritable traits may shape the environment by evoking specific reaction of others and hence the environment provided by others [29] have been studied in mother-child relationships [33]. For instance, a mother with separation anxiety may be creating a unique environment around her by virtue of her ASA and this adverse environment may in turn lead to specific maternal behaviors in response to her infant; hence we tested evocative gene-environment interactions using ASA as the environment. Our results supported a significant G × E association between OXTR cumulative genetic risk score and assignment to high and low adult separation anxiety on observed maternal sensitivity during the free play stage of the SFP, such that mothers with a higher cumulative genetic risk score and with high separation anxiety showed significantly decreased levels of maternal sensitivity compared to mothers with higher cumulative genetic risk score and low separation anxiety. Building upon the published cumulative genetic risk score, we calculated an extended OXTR genetic risk score by adding in the risk information from rs968389, an independent SNP locus which we found to be implicated in maternal oxytocin response and maternal separation anxiety from the infant. Using this extended cumulative genetic risk score, we replicated the G × E association between OXTR cumulative genetic risk score and adult separation anxiety (high versus low) on observed maternal sensitivity during free play. Furthermore, we identified that the extended OXTR cumulative genetic risk score itself was significantly associated with maternal separation anxiety from the infant; however this association was driven specifically by rs968389. These results suggest a complex mosaic of individual and synergistic genetic effects of OXTR on different aspects of maternal behavior.

While several interesting and novel findings emerge from this study, there are some limitations. The small sample size and candidate-based approach suggest that it would be important to replicate these findings in larger samples while also performing an unbiased genome-wide scan to identify polygenic predictors of maternal oxytocin response and maternal behavior. However, the use of a high-risk targeted sample such as the one used for the current study might present an adequate target for highly specialized analysis targeting G × E interactions in well-defined loci.

In summary, we identify a novel SNP, rs968389, that is associated with maternal oxytocin response and maternal behavior. These results contribute to a better understanding of the neurobiological underpinnings of oxytocin and its role in maternal and infant behavior with consequent implications for mother-infant interactions and potential therapeutic interventions that would merit further research.

## Supplementary Material

The publicly available GTeX database [31] and the Braineac database of the UK Brain Expression Consortium () revealed that rs968389 was significantly associated with gene expression of the OXTR gene within the caudate (basal ganglia) brain tissue (p-value = 1.7E-8) as well as the frontal cortex (*p*-value =8.7E-7) among others, providing evidence of functional consequences of the SNP (Supplementary Figures 1a and 1b).

## Figures and Tables

**Table 1:** Demographic details of the 96 women included in the study.

Variable	Mean [SD]/*N* [%]
Age	29.90 [0.57]
Ethnicity:	
Caucasian	46 [47.9%]
Asian	32 [33.3%]
Arab	12 [12.5%]
Other	6 [6.3%]
Adult separation anxiety:	
Yes [≥22]	48 [50%]
No [<21]	48 [50%]
rs968389 genotype:	
AA	29 [30.2%]
AG	36 [37.5%]
GG	30 [31.3%]
