# Supplementary material for: Genetic Regulation of Maternal Oxytocin Response and Its Influences on Maternal Behavior
Source: Neural Plast. 2016 Oct 31;2016:5740365. doi: 10.1155/2016/5740365 (PMC5107871; doi:10.1155/2016/5740365)
Supplement: Supplementary file 1 — The publicly available GTeX database [31] and the Braineac database of the UK Brain Expression Consortium () revealed that rs968389 was significantly associated with gene expression of the OXTR gene within the caudate (basal ganglia) brain tissue (p-value = 1.7E-8) as well as the frontal cortex (p-value =8.7E-7) among others, providing evidence of functional consequences of the SNP (Supplementary Figures 1a and 1b). [file 5740365.f1.pdf]

Supplementary Figure 1a

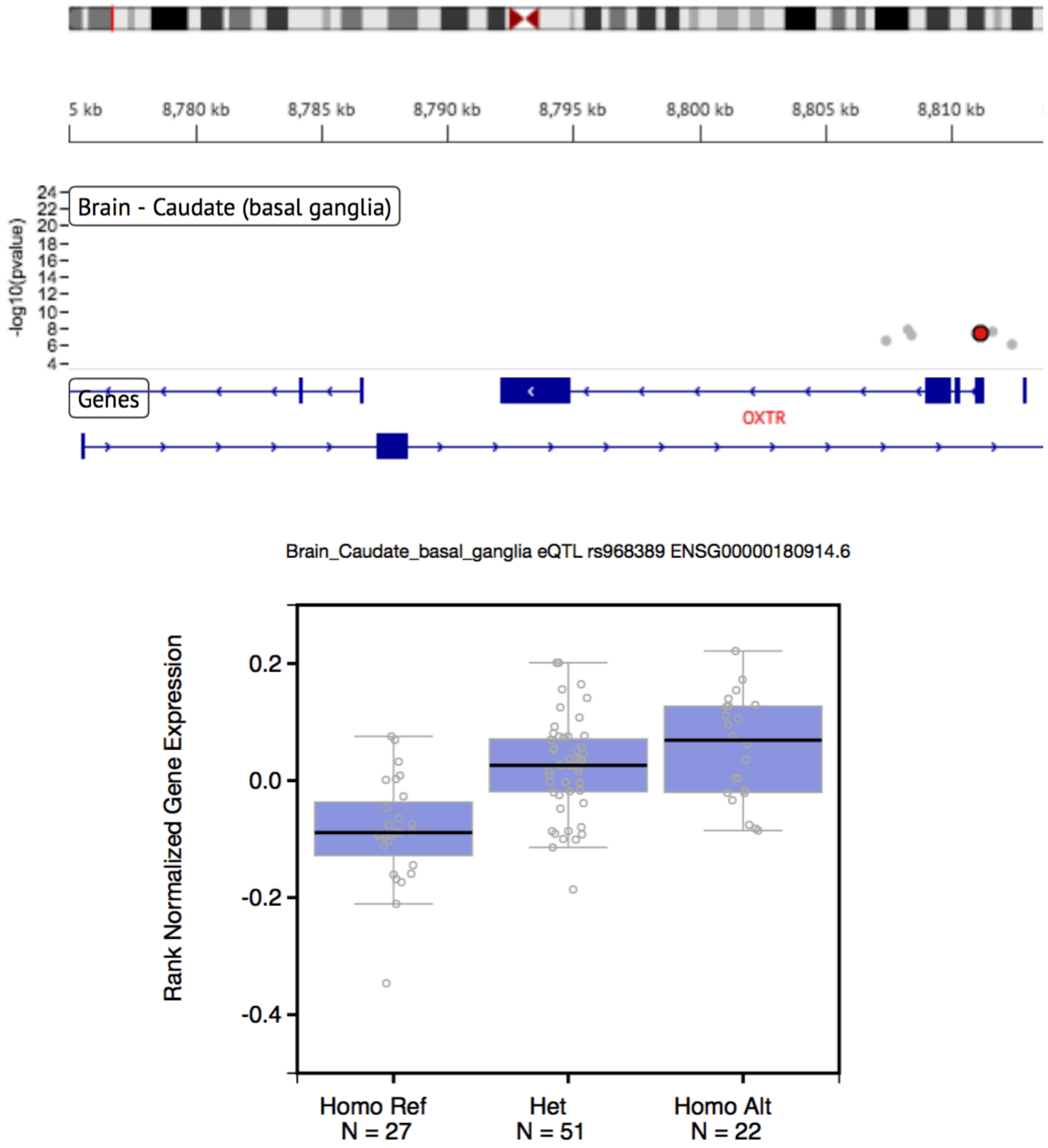

Supplementary Figure 1a: rs968389, located in the 5'UTR of the OXTR genes, is significantly associated with gene expression levels of OXTR gene in caudate basal ganglia in the brain, using the GTEX database [31]. Homo Ref refers to the AA genotype group, Het refers to the AG and Homo Alt refers to the GG genotype group.

Supplementary Figure 1b

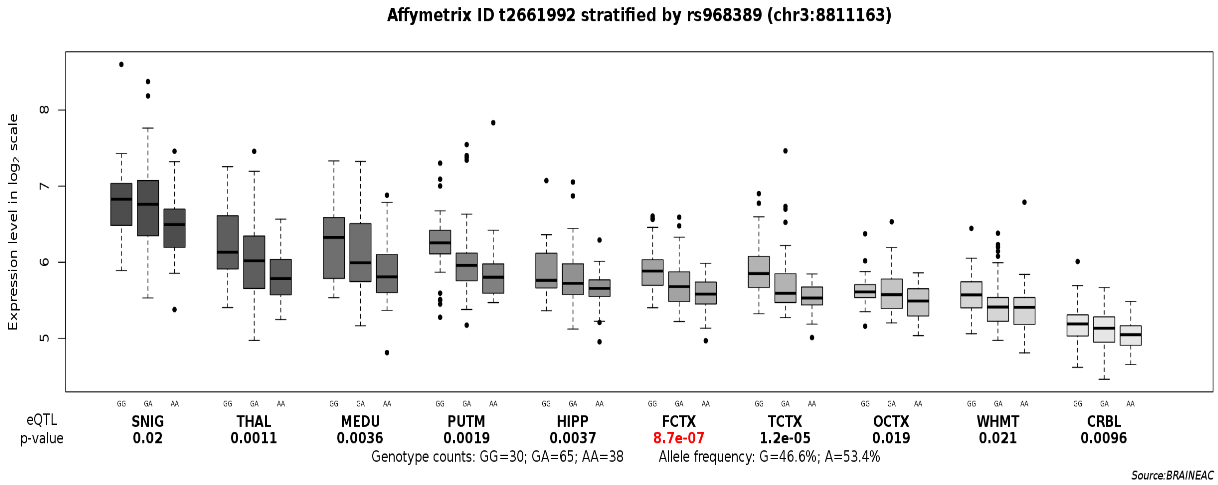

Supplementary Figure 1b: Using the Braineac database (http://www.braineac.org/) from the UK Brain Expression Consortium, rs968389 was significantly associated with gene expression levels of OXTR gene in 10 brain regions: cerebellar cortex (CRBL), frontal cortex (FCTX), hippocampus (HIPP), medulla (specifically inferior olivary nucleus, MEDU), occipital cortex (specifically primary visual cortex, OCTX), putamen (PUTM), substantia nigra (SNIG), thalamus (THAL), temporal cortex (TCTX) and intralobular white matter (WHMT). Red denotes the frontal cortex region where the SNP showed the strongest association with gene expression. The risk genotype GG was associated with increased gene expression of the OXTR gene in the brain regions.
